# Aluminium in Biological Environments: A Computational Approach

**DOI:** 10.5936/csbj.201403002

**Published:** 2014-03-28

**Authors:** Jon I Mujika, Elixabete Rezabal, Jose M Mercero, Fernando Ruipérez, Dominique Costa, Jesus M Ugalde, Xabier Lopez

**Affiliations:** a Kimika Fakultatea, Euskal Herriko Unibertsitatea (UPV/EHU), and Donostia International Physics Center (DIPC), P.K. 1072, 20080 Donostia, Euskadi, Spain; b Laboratoire de Chimie Moleculaire, Department of Chemistry, Ecole Polytechnique and CNRS, 91128 Palaiseau Cedex, France; c POLYMAT, Euskal Herriko Unibertsitatea UPV/EHU. Joxe Mari Korta zentroa, Tolosa Etorbidea 72, 20018 Donostia-San Sebastián, Euskadi, Spain; d Laboratoire de Physico-Chimie des Surfaces (UMR 7045), ENSCP Chimie-Paristech, 11 rue P. et M. Curie, 75005 Paris, France

## Abstract

The increased availability of aluminium in biological environments, due to human intervention in the last century, raises concerns on the effects that this so far “excluded from biology” metal might have on living organisms. Consequently, the bioinorganic chemistry of aluminium has emerged as a very active field of research. This review will focus on our contributions to this field, based on computational studies that can yield an understanding of the aluminum biochemistry at a molecular level. Aluminium can interact and be stabilized in biological environments by complexing with both low molecular mass chelants and high molecular mass peptides. The speciation of the metal is, nonetheless, dictated by the hydrolytic species dominant in each case and which vary according to the pH condition of the medium. In blood, citrate and serum transferrin are identified as the main low molecular mass and high molecular mass molecules interacting with aluminium. The complexation of aluminium to citrate and the subsequent changes exerted on the deprotonation pathways of its tritable groups will be discussed along with the mechanisms for the intake and release of aluminium in serum transferrin at two pH conditions, physiological neutral and endosomatic acidic. Aluminium can substitute other metals, in particular magnesium, in protein buried sites and trigger conformational disorder and alteration of the protonation states of the protein's sidechains. A detailed account of the interaction of aluminium with proteic sidechains will be given. Finally, it will be described how alumnium can exert oxidative stress by stabilizing superoxide radicals either as mononuclear aluminium or clustered in boehmite. The possibility of promotion of Fenton reaction, and production of hydroxyl radicals will also be discussed.

## I. INTRODUCTION

Aluminium is the most abundant metal element on the Earth crust, however, biological systems have evolved in the absence of this abundant metal. This apparent paradox can be understood in terms of the effective geo-chemical control of aluminium by means of its interaction with silicic acid [1]. Other metal ions such as Mg(II), Fe(II)/Fe(III), Ca(II), Zn(II) etc, have been biologically available, and biological systems have evolved in the presence of these metals, coordinated to phosphate, carboxylate, hydroxyl and other ligands. However, in the last century, human intervention has made aluminium, sparingly soluble, so available for biological systems that one can say that we have started to live in the aluminium age. However, little is still known on the effects of the human exposure to this element, although one could suspect that its effects should be important due to the highly charged nature of aluminium. In fact, in the last years, there are increasing evidences that aluminium could be behind of a variety of toxic effects in biological systems [2–4], with significant risks for human health. Therefore, the open of the geochemical pandora-box of aluminium into biological systems is unlikely to be without consequences.

The aluminium speciation problem, that is the characterization of the type of aluminium complexes likely to be formed in biological medium, is a complex problem, due in part to the vast variety and complexity of aluminium hydrolytic species [5], their low solubility and their spectroscopic silence. In this sense, computational methods have become a fundamental tool to understand aluminium speciation in biological systems and determine the characteristics of aluminium interaction with molecules of biological interest. With no claim of being complete, we can list four connected areas in which computation can help to unveil specific details of aluminium-ligand interactions:

- Characterization of aluminium interaction with biomolecular building blocks: amino acids, phosphates, etc, so that fundamental understanding of the intrinsic affinity of aluminium for functional groups representing the building block motifs of biomolecules can contribute to the elucidation of aluminium binding sites in biological systems. In addition, comparison of these affinities with those of essential biometals can help in understanding the propensity for displacement of a given metal by aluminium.
- Determination of the various aluminium hydrolytic species that could be formed in aqueous solution as a function of pH implies the study of various protonation states, tautomers and oligomers that aluminium can form in solution [6–9].
- Interaction of aluminium with high molecular weight (HMW) ligands such as proteins, is central for the determination of aluminium speciation in blood. In this sense, serum transferrin is one of the most important blood aluminium carriers. Besides, interaction with β-amyloids have also been identified, and it could be behind the controversial role of aluminium in neurodegenerative diseases.
- Interaction of aluminium with low molecular weight (LMW) species commonly present in biological media, could play a role in its transport and fixation in solution. These molecules normally contain various carboxylate-type functional groups in the same unit. Oxalate and citrate are examples of this type of molecules. Besides, interaction with LMW ligands could also be behind some of its most relevant toxic effects. Namely, it has been recently pointed out that aluminium can be involved in the stabilization of superoxide complexes [10] that trigger the Fenton reaction [11].

All these areas are interconnected, for instance to characterize the mode of interaction of aluminium with proteins (Section IV B), it is important to understand first the interaction with amino acid sidechains (Section II), and how aluminium affects the pK_a_ of these amino acids (Section III). The characterization of the protonation/deprotonation equilibria is fundamental to understand how aluminium chelates low molecular weight ligands (Section IV A) and high molecular weight proteins (Section IV B). In addition, the analysis of aluminium hydrolytic species is key to understand changes in affinities of aluminium with respect to ligands (Section V A), and some of these interactions could be behind the toxic effects of aluminium (Section V). In the present review, we give examples of how computational studies can assist in each of these areas.

The calculations can shed light on the type of aluminium species that one could find in aqueous environment, and the affinity of aluminium species towards common biomolecules. In addition, calculations can also shed light on the effect that a highly charged metal such as Al(III) could have in the structure of biomolecules bound to this metal. Herewith, we give a number of selected examples of how computational methods can be used to unveil some of the essential characteristics of aluminium interaction with biological systems, and in this sense, help in the understanding of the hazards that living in the aluminium-age could have for biology.

## II. ALUMINIUM INTERACTION WITH BIOMOLECULAR BUILDING BLOCKS: PROTEIN ENVIRONMENTS

Understanding the interaction of aluminium with biological building blocks is essential for the determination of the effect of aluminium in biological systems. The most interesting building blocks with respect to aluminium interaction are amino acid side chains commonly present in metal-ion binding sites, and phosphates ubiquitously present in DNA, RNA, ATP, etc [12]. A first step towards this goal in the group was carried out by Mercero [13–18] and then Rezabal [19–21], who analyzed a series of clusters in which aluminium interacts with various amino acid sidechains in a proteic environment. The protein environments were modeled with the so-called cluster-continuum approach [22, 23]. In this approach, we consider different molecules representing the amino acid sidechains (acetate as a representative model for glutamate and aspartate, methyl-thiol/thiolate for cysteine, methylthioethane for methionine, acetamide for asparagine and glutamine, methanol for serine and threonine, methylimidazole for histidine, and toluene and methylbenzenol for phenylalanine and tyrosine respectively) chelating the metal, and the rest of the octahedral first-coordination shell around aluminium is filled with water molecules. The chosen ligands do not only represent the metal binding site in a protein, but also other organic molecules present in the biological systems, taking part in aluminium metabolism. The whole cluster, considering various combinations and different number (1 to 3) of ligands, is then surrounded by a continuum dielectric to represent different proteic environments, from protein buried sites (small dielectric values ɛ=2, 4, …) to fully solvent exposed areas (high dielectric values ɛ=80). The results were compared to analogous Mg(II) clusters.

### A. Metal binding Affinity

The metal binding affinity was evaluated by calculating the energy of the following reaction:

L_m_^q^+X(H_2_O)_6_^ch^ → XL_m_(H_2_O)_(6-m-n)_^(ch+q)^+(m+n)H_2_O

where ch and q are the charge of the metal cation and the sum of the charges of the m ligands, respectively, n corresponds to the number of ligands (acetates) bound bidentately, and X stands either for the Al(III) or the Mg(II) cations. The reaction defines the metal binding affinity as the water/ligand substitution from the first hydration shell of the metal, where all the exchanges occur simultaneously. It was observed that both Al(III) and Mg(II) share ligand preferences, favoring binding to oxygen and nitrogen groups, in particular negatively charged oxygens. Therefore, the negatively charged acetate and the neutral methylimidazole, followed by formamide and methanol were seen to be preferred for binding Al(III). The monodentate binding mode of acetate was stabilized as compared to the bidentate mode, due to the interaction of the metal-free carboxylate oxygen atoms with the metal-bound water molecules. The binding of the metals to the bioligands was found to be mainly dictated by the favorable Coulomb interactions between the positively charged cation and the negatively charged or neutral ligands, and the solvation free energies of the products and reactants in the dielectric environment considered. Al(III), due mainly to its high charge, has a strong tendency of binding these bioligands, but its desolvation free energy is also very high. The delicate balance between the charge and number of ligands and the dielectric environment regulates the affinity of the metal for the binding sites.

Therefore, we stablish that aluminium will prefer to bind proteins (low dielectric environment) rather than small low weight molecules, in an aqueous environment. Nevertheless, the formation of aluminium complexes in both gas and aqueous phases is promoted when the number of available ligands increases. In particular, binding sites with two acetates or three ligands (at least one of them being an acetate) were energetically favored to bind aluminium in the whole range of dielectric constants. In fact, these kind of multidentate ligands with negative oxygen donors are known to be the best chelators for Al(III) [24]. The main example is citrate, which, with three donor groups bound to aluminium, is the main low weight molecule which carries aluminium in blood [25]. In buried protein zones Al(III) has been seen to attach binding sites with only one ligand, but, still, the preferred binding sites are those presenting three ligands, one of them being acetate. This behavior parallels that of natural Mg(II) binding sites; in fact, numerous examples of Al(III) inhibition of Mg(II) dependent metalloenzymes have been reported [26]. Both cations are of similar size, a factor that dominates over the charge identity towards metal competition [27–30] suggesting that Al(III) should seek the sites normally served by Mg(II) [27]. In order to explore this hypothesis, the Al/Mg exchange reaction was studied in more detail considering both metals having the same surrounding ligand environment.

### B. Metal Exchange Reaction

The metal exchange reaction was defined as follows:

MgL_m_(H_2_O)_6-m-n_^2+q^ + Al(H_2_O)_6_^3+^ → AlL_m_(H_2_O)_6-m-n_^3+q^ + Mg(H_2_O)_6_^2+^

The energy balance of this reaction, the exchange energy, indicates the likelihood for the hydrated Al(III) to substitute Mg(II) already attached to a binding site in a protein. Two different situations were considered: (1) the exchange occurs in the environment set up by the protein cavity, or (2) the incoming metal arrives directly from solution and the replaced one goes to solution (thus the dielectric constant for the hydrated cations environment will always be that of water). The displacement reaction was observed to be driven by the balance of the relative Coulombic interactions of the metals with the negatively charged ligands in the site, and the desolvation penalty of the charged reactants as compared to the solvation energy gain of the non charged or less charged products. Desolvation in the first scenario (Fig. 2) consists of the removal of the cation's hydration shell, while in the latter (Fig. 3) includes also the extraction of the hexahydrated moiety from the aqueous environment. Al(III), due to its larger charge, sets stronger Coulomb interactions, but has a considerably higher desolvation penalty than Mg(II). Consequently, Al(III) presents strong favorable thermodynamical propensity to substitute Mg(II) in proteins, even if the metal exchange is somewhat constrained in the most solvent exposed areas. In this case, exchange would be restricted to binding sites having two negatively charged ligands.

**Figure 1:**
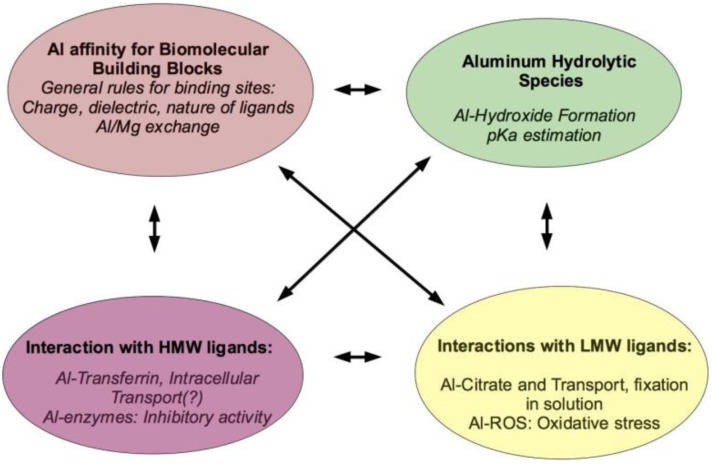
Understanding the problem of aluminium speciation in biology requires the interplay between different areas.

**Figure 2:**
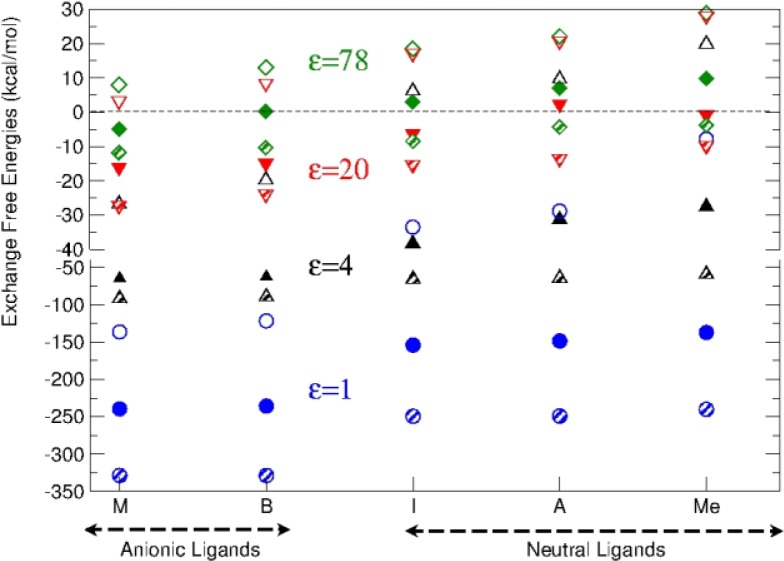
Metal exchange reaction free energies for selected dielectric constant values. Circles stand for fully buried sites (ε = 1), diamonds for fully solvent exposed sites (ε = 78) and the up triangles and down triangles for the dielectric constant values 4 and 20, respectively. The hollow symbols correspond to the single ligand complexes, and the filled symbols to the two ligand complexes, where one ligand always corresponds to a monodentate acetate, and the second is denoted on the x-axis. Finally, the striped symbols denote the complexes with two monodentate acetates together with the ligand indicated on the x-axis. Notice that the energy scale changes at -40 kcal/mol.

**Figure 3:**
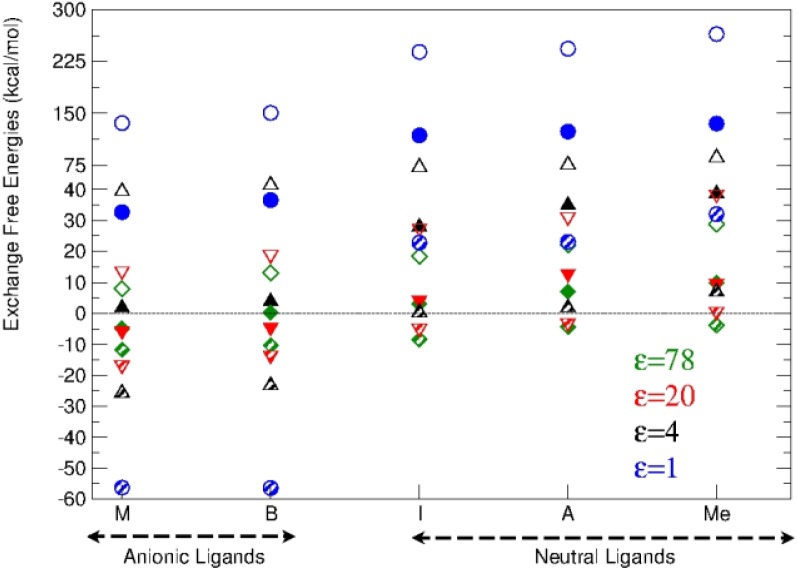
Metal exchange reaction free energies for selected dielectric constant values. Circles stand for fully buried sites (ε=1), diamonds for fully solvent exposed sites (ε=78) and the up triangles and down triangles for the dielectric constant values 4 and 20, respectively. The hollow symbols correspond to the single ligand complexes, and the filled symbols to the two ligand complexes, where one ligand always corresponds to a monodentate acetate, and the second is denoted on the x-axis. Finally, the striped symbols denote the complexes with two monodentate acetates together with the ligand indicated on the x-axis. Notice that the energy scale changes at 40 kcal/mol.

In the second scenario proposed, this balance was more subtle, and the substitution ability of Al(III) was strongly impaired due to the desolvation penalty. Interestingly, it was observed that the most frequent binding sites served by Mg(II), namely three carboxylates or two carboxylates and one neutral ligand, fulfill the conditions for the substitution to be thermodynamically favored in the environmental conditions taken into account. Among the Mg(II)-dependent enzymes inhibited or altered by aluminium, some of them as acetilcholinesterase, alkaline phosphatase, or adenylate cyclase, fulfill this conditions, rendering the substitution possible from the thermodynamical point of view.

## III. ALUMINIUM DRIVEN SHIFT OF THE pK_a_ OF SELECTED AMINO ACIDS

An important aspect of Al(III) interactions with amino acids is the shift that this metal can provoke in the sidechains forming the metal ion site. This could lead to an alteration of the protonation state of residues directly coordinated to Al(III), with the concomitant effects on the structure and activity of a given metalloprotein. In this section, we show how theoretical calculations can help in the evaluation of the shift in the pK_a_ of selected amino acids, mainly oxygen containing ones, when interacting with Al(III).

**Figure 4:**
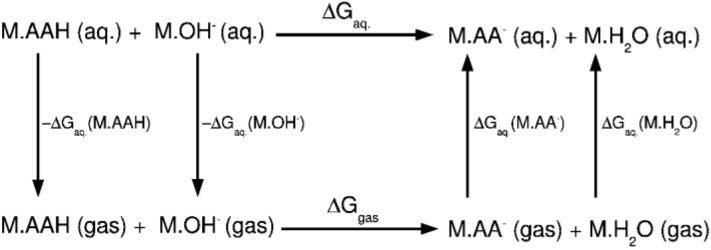
Thermodynamic cycle used to calculate relative pKa's for Al(III)/Mg(II)-amino acid systems. The relative pK_a_ is calculated with respect to a water molecule bound to the metal.

Several works have been published estimating the absolute pK_a_ of wide variety of molecules with reasonable success [31–34]. Computationally, the evaluation of a pK_a_ is not exempt from difficulty and diverse approaches have been employed for an accurate evaluation of pK_a_, which are summarized in several reviews [35, 36]. In principle, the evaluation of an absolute pK_a_ would require the accurate estimation of the solvation free energy of H^+^. However, depending on the experiment, the solvation energy of H^+^ can differ in ca. 5 kcal/mol, which may suppose a deviation of 3 units in the final pK_a_ value. An alternative of the absolute or direct evaluation of pK_a_ is the evaluation of a relative pK_a_ with respect to a molecule, for which the pK_a_ is experimentally known. Thus, one considers the deprotonation of the acidic group as a proton transfer to a second molecule, preferably a water molecule. This strategy, schematized in Fig. 4, avoids the treatment of the solvation energy of the proton, and it has been employed in many studies with satisfactory results [23, 37, 38].

Another aspect of the work is that one needs to consider improved cluster-continuum models, in which, as suggested by Yang et al.[39], there must be included at least two explicit solvation layers around the aluminium atom. As it was demonstrated, this type of models can yield accurate thermodynamics of deprotonation, and therefore, accurate pK_a_ values. The accuracy of our protocol was tested by comparing the experimental and computational pK_a_ of a water molecule interacting with Al(III), considering an isolated hydroxide molecule as the basic molecule. If only the first solvation sphere of the hydrated Al(III) complex is considered, a poor result is obtained. However, when the second solvation sphere is explicitly treated by adding twelve water molecules, the value of the pK_a_ improves, yielding a value of 4.6, in very good agreement with the experimental value of 5.0 and strongly supports the results of Yang et al [39]. Therefore, inclusion of explicit water molecules at the second hydration sphere was seen to be key to yield accurate pK_a_ evaluations.

**Figure 5:**
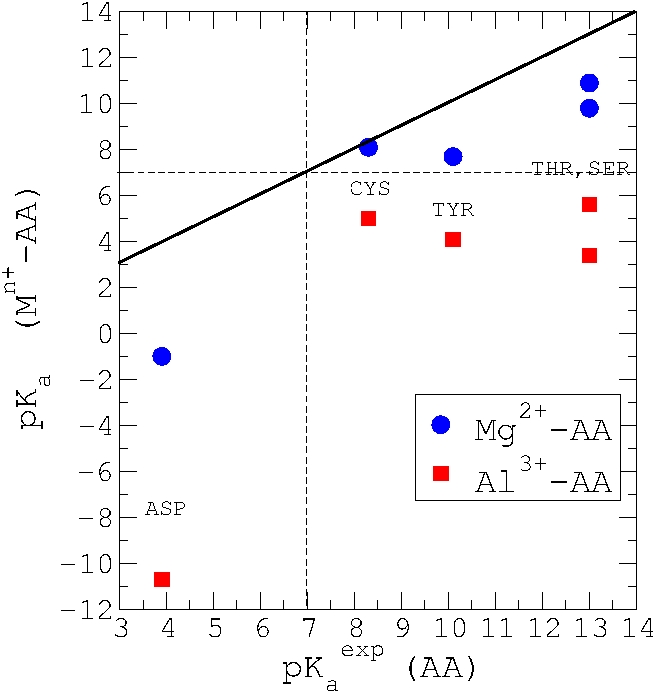
pK_a_ shifts caused by Al(III)/Mg(II) in amino acid (AA) sidechains representing Asp, Cys, Tyr, Thr and Ser. Contrary to Mg(II), we predict that Al(III) is able to deprotonate all these residues at physiological pH's.

Using this protocol, the pK_a_ of amino acids with an acidic OH group were included in the study, that is, Asp, Tyr, Ser and Thr. Besides, these amino acids are among the most prone ones to Al(III) interactions, as we described in the previous section. Due to the chemical similarity between oxygen and sulfur, Cys was also studied. The results are summarized in Fig. 5. In order to compare the differential effect of Al(III) insertion in a metal ion site, we have decided to re-calculate the pK_a_'s for a metal such as Mg(II). From our results, it is clear that Al(III) has a big influence on the acidity of these amino acids, and we can predict important shifts in the pK_a_ of these amino acid side chains when coordinated to Al(III). In particular, our data suggests that Asp would show the largest pK_a_ drop, going from 3.9 units in solution to -10.7 when interacts with Al(III). The other amino acids show also much lower pK_a_ values: Tyr from 10.1 to 4.1, Ser from 13.0 to 3.4, Thr from 13.0 to 5.6 and Cys from 8.3 to 3.3. Our results also confirm the idea that the interaction of these residues with Al(III) could provoke a change in the protonation state of the neutral residues treated in this work (Tyr, Ser, Thr, and Cys), since all of them show pK_a_'s lower than typical physiological pH values upon interaction with Al(III). The chemical importance of such shift should not be underestimated, since a change in the protonation state of a given amino acid can lead to important changes in the structure and consequently in function of proteins in which Al(III) would be inserted.

When Mg(II) is considered, there is also, in general, an increase in the acidity of these residues (lower pK_a_'s), but the lowering of these pK_a_'s is significantly less pronounced than in the Al(III) case: Asp (-1.0), Tyr (7.7), Cys (8.1), Ser(9.8-13.6) and Thr (10.9-14.8). However for the amino acids that are neutral at standard conditions (Tyr, Cys, Ser and Thr) the shift in their pK_a_'s upon interaction with Mg(II) is not sufficient as to become deprotonated at physiological pH. This is in contrast to the behavior highlighted above for Al(III), and pinpoints to a major effect of Al(III)/Mg(II) substitution at the Mg(II) metal binding sites. Therefore theoretical methods contributes to the understanding of the effect of aluminium in the protonation equilibria of amino acid sidechains. This protonation/deprotonation of bioligands by aluminium plays also a very important role for the metal chelation by bioligands in blood. Next we show two examples: citrate and serum transferrin, the two major chelators of aluminium in blood.

## IV. ALUMINIUM SPECIATION IN BLOOD: COMPLEXATION WITH CITRATE AND TRANSFERRIN

As it was indicated in the Introduction section, aluminium has been linked with several diseases. In order to understand the toxic effects of aluminium, the speciation of this element in blood serum, that is, a knowledge of the biological molecules interacting with the metal in blood serum is necessary. However, this is a difficult task due to its complex chemistry, its low total concentration and the high risk of contamination [40]. The bioligands that prefer to form stable complexes with aluminium in serum have been classified as high molecular mass (HMM) proteins and low molecular mass (LMM) molecules. The group of Milacic demonstrated [41] that in blood serum transferrin (sTf) is the main HMM species bound to Al(III), while citrate is the main LMM species. Recently, Beardmore and Exley pointed out [42, 43] that to understand the dynamics of Al(III) in blood serum one should also take into account the non-equilibrium binding of Al(III) to several other ligands. This can be done using a “system-biology approach” computational model [42].

Due to the fact that most of the aluminium in blood serum is bound to serum transferrin protein, initially it was assumed that aluminium follows the iron pathway to enter the cell [44, 45]. However, experiments have shown that the aluminium-loaded sTf has lower affinity towards transferrin receptor (TFR) [46, 47]. Therefore, it has been suggested that aluminium may follow other paths to get into the cell. In this sense, Yokel et al. proposed [48] that aluminium can get into the brain complexated to citrate, presumably mediated by putative monocarboxylate transporter [49, 50]. In fact, the amount of aluminium bound to citrate is significantly larger in cerebrospinal fluid than in serum [51].

Even that the pathway followed by aluminium to get access into the cells is not entirely understood, it is evident that aluminium shows preference for interacting with serum transferrin protein and citrate. Furthermore, the knowledge about these interactions at an atomistic level is still scarce due to the complex intrinsic characteristics of aluminium. In this vein, the information provided by computational chemistry can be determinant to further understand how Al(III) interacts with its main bioligands in blood serum. In the next subsection we summarize the studies carried out by our group on Al(III) interacting with citrate and serum transferrin.

### A. Al complexation with citrate and its effect on the deprotonation pathway

**Figure 6:**
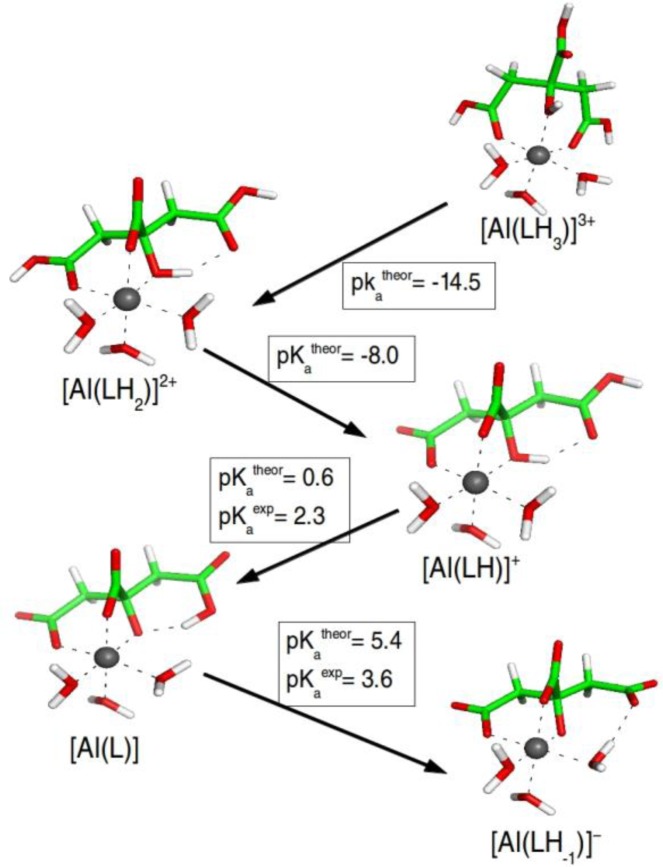
Most stable conformation for each of the protonation states of citric acid interacting with Al(III). The computational pK_a_ values of the citric acid interacting with Al(III) are shown and compare with available experimental values taken from ref [52].

Citric acid is composed of two terminal and one central carboxylic groups, and a central hydroxyl group (see Fig. 6). The molecule contains four O-containing groups that are ionizable and suitable as aluminium coordination sites. Due to steric effects only three of the four binding sites can interact with Al(III). The stechiometry of the complex has extensively been studied. Al(III) can be complexated with one or two citric molecules, and complexes with more than one Al(III) cation can also be formed. Time-dependent potentiometric measurements [53] indicated that in the 3-7 pH range two types of 1:1 mononuclear species are predominant in solution, [Al(LH_-1_)]^-^ and [Al(OH)(LH_-1_)]^2-^ (nomenclature presented in Fig. 6). The difference between these two species lies on the protonation state of the citric acid. Similarly, in another study combining time-dependent potentiometric and NMR spectroscopic measurements [52], various species with 1:1 and 1:2 stechiometry were formed. Among them, the neutral [Al(L)] complex is of special interest, because this species is thought to pass through membranes [27]. For the [Al(LH)]^+^ complex, NMR spectroscopy unambiguously determined that the binding mode of the citrate involves a terminal carboxylic group, the central carboxylic group and the hydroxyl group [52]. The same binding mode is present in the crystal structure solved for the mononuclear [Al(L)_2_]^3-^ [54] and [Al(L)(LH)(LH_-1_)]^4-^ [55] species. Therefore, the complexation of citrate to aluminium led to the formation of multiple species with a variety of protonation states. The experiments provide information about which complex is formed at different conditions, but nevertheless they do not clarify the protonation states presented by each donor group of citric acid. Moreover, since the experiments were carried out at mild pH conditions (in the 2-8 pH range), not all Al(III)-citrate species have been captured, what limits severely the knowledge about the deprotonation process. The main aim of our study [56] was to analyze the deprotonation process of citric acid in solution (i.e, without the presence of Al(III)) and chelated to Al(III), what allowed us to determine the influence of Al(III) onto the citric acid's acidity. To do so, all possible protonation states of the citric acid were considered and when this molecule was interacting with Al(III), all binding modes were taken into account. Once the most stable tautomers for each protonation state of citric acid were stablished, the pK_a_ values of all titratable groups of citric acid in solution and complexated to Al(III) were evaluated, using a similar cluster-continuum model as the ones explained in Section III.

The results (shown in Fig. 6) showed that the interaction of the citric acid with aluminium has a big influence on its deprotonation process and acidity, and that the coordination mode of the molecule is a key factor to understand the deprotonation process. Due to the coordination mode of citrate to Al(III), the order in which the ionizable groups of the citrate are deprotonated varies with respect to the free molecule. Thus, in the two systems the central carboxylic group is the first group being ionized, followed by one of the terminal carboxylic groups. However, while in the free citrate the other terminal carboxylic group is the next group being deprotonated, upon chelation to Al(III) the hydroxyl group is deprotonated first, and then the carboxylic group. This change in the order is due to the coordination mode of citrate to Al(III), favoring the ionization of the Al(III)-bound hydroxyl group rather than the deprotonation of the carboxylic group not interacting with the cation. Comparing the pK_a_ values computed for the free citric acid and interacting with Al(III), we could predict the shifts in the pK_a_. The pK_a_ values of the central carboxylic group and the first terminal carboxylic group decreased from 1.4 to -14.5 and from 4.9 to -8.0, respectively, when they were coordinated to the cation. The pK_a_ of the hydroxyl group decreased from 10.2 to 0.6 (the third pK_a_ value). Thus, the pK_a_ of these three groups decreased in 15.9, 12.9 and 9.6 units, respectively. Note that a drop of 10-15 units of pK_a_ was also observed in the previous section for amino acids interacting with Al(III)[57]. On the other hand, the acidity of the second terminal carboxylic group did not vary much and went from 5.2 in the free citrate to 5.4 in the Al(III)-citrate complex. In fact, our results are also coherent with the X-ray crystal structure for the [Al(LH_-1_)]^-^ species [55], where an unprotonated hydroxyl group and protonated carboxyl groups were determined.

### B. Al(III) complexation with serum transferrin: the role of pH and protonation of Tyr188

**Figure 7:**
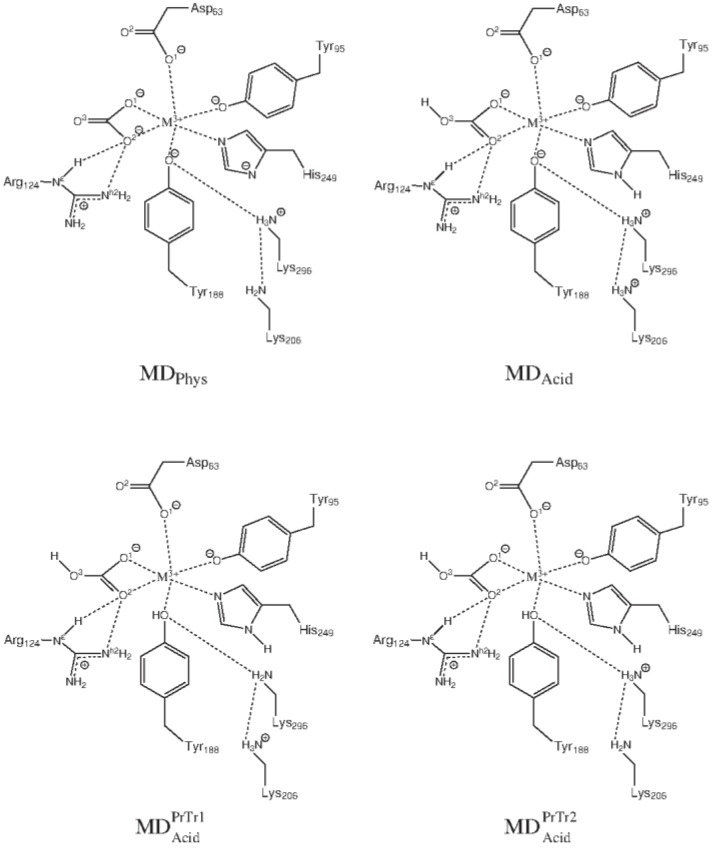
Schematic representation of the transferrin metal (M=Fe(III) or Al(III)) binding site for four systems: MD_Phys_, MD_Acid_, MD_Acid_^PrTr1^ and MD_Acid_^PrTr2^.

**Figure 8:**
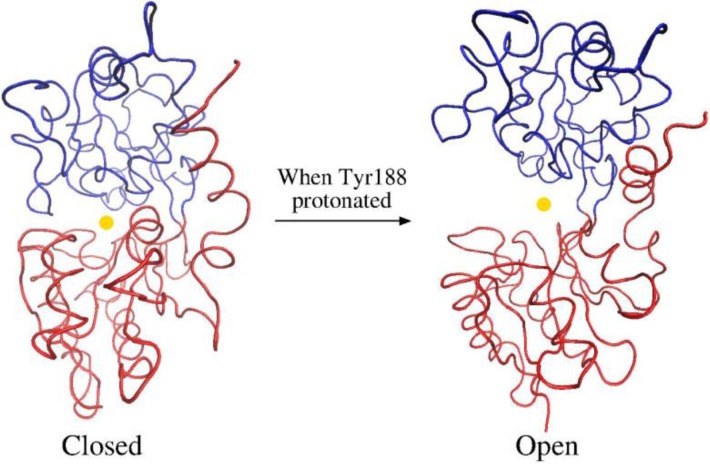
The two conformation adopted by the metal-loaded serum transferrin. The protein only opens in those MD simulations^a)^ with Tyr188 protonated.

As we will show in this subsection, protonation/deprotonation of residues directly interacting with Al(III) plays also a very significant role in the intake and release mechanisms of aluminium in serum transferrin (sTf). Several X-ray structures of the aluminium-loaded Tf have been solved [58], and experiments using X-ray absorption near edge structure (XANES) spectroscopy shows that aluminium ion is hexacoordinated in the complex, presenting a octahedral-like symmetry [59]. However, there is not information about the metal coordination mode once the complex is introduced into the endosome. sTf has a chain folded into two globular lobes (N- and C-lobes) connected by a short protein linkage. Each lobe contains a metal binding site set up by two subdomains connected by a hinge, forming a cleft where the metal can be placed. In both the C- and N- binding sites (Fig. 7), the metal is coordinated by an aspartic acid, two tyrosines and a histidine. X-ray crystal structures of the transferrin family members determine that the transferrin protein presents two different conformations (represented in Fig. 8), an open conformation when it is metal free [60], and a closed conformation upon the binding of Fe(III) [61]. It is accepted that the conformational change upon the metal release process involves two global motions: hinge-twist and hinge-bending [62].

It was suggested that Lys206 and Lys296 residues modulate the opening of the metal loaded protein by forming the so-called “dilysine trigger”. These two residues form a hydrogen bond interaction in the iron-loaded protein [63], while the distance between them is significantly larger in the apoform [60]. This difference was explained by a different protonation state of Lys206, neutral at physiological pH but protonated at the endosomal pH of 5.5 [64]. Nevertheless, some authors argued that the dilysine trigger alone cannot explain the metal release process [64, 65], and suggested that the protonation of Lys206 at the endosomal pH prompts the protonation of Tyr188 by Lys296. Thus, protonation of Tyr188 is needed for the metal release, and for this protonation to happen a low pH is needed. This hypothesis is reinforced by our estimations of a low pK_a_ for the Al(III)-bound tyrosine (Section III). In addition, analysis of the ^13^C chemical shift in apo-hTF/2N also pinpoints [66] to a significant shift of the pK_a_ of this tyrosine.

Our research focused into two main points: on one hand, we investigated whether the protonation of Tyr188 is required to prompt the opening of the protein previous to the metal release [67], and on the other hand, we analyzed more specifically the interaction mode of aluminium and iron to sTf at different pH conditions [68].

#### 1. Molecular Dynamics Simulations

In order to investigate the consequences of protonating Tyr188 [67], a total of eight molecular dynamics simulations were carried out for the complexes Fe(III)-sTf and Al(III)-sTf. For each of these two complexes, four protonation states were considered (shown in Fig. 7). In MD_Phys_ system, the protonation states of the amino acids were adjusted to the pH conditions in blood serum (pH=7.4), so that Lys206 remains in its neutral protonation state. In the rest of the simulations, MD_Acid_, MD_Acid_^PrTr1^ and MD_Acid_^PrTr2^, the more acidic pH found in the endosome was considered, which modifies the protonation states of some amino acids. In the MD_Acid_ system, both Lys206 and Lys296 are protonated, while Tyr188 remains unprotonated. If the dilysine trigger explanation is enough to explain the metal release process, an opening of the protein should be observed during the simulations due to the repulsion between the two positively charged lysine residues. On the other hand, in the MD_Acid_^PrTr1^ and MD_Acid_^PrTr2^ system Tyr188 has been protonated. Since this proton comes from one of the two lysines, the difference between these two systems relies on which lysine is unprotonated and which remains protonated.

During the two simulations with the protonation state at physiological conditions, i.e., the MD_Phys_ system with sTf loaded with Al(III) or Fe(III), the protein showed equivalent conformations. The protein adopted a conformation similar to the one presented at the structure solved by X-ray diffraction: a closed conformation with a stable hydrogen bond interaction between Lys206 and Lys296. Similarly, in the MD_Acid,Fe_ and MD_Acid,Al_ simulations, even that Lys206 and Lys296 bore a positive charge and the electrostatic repulsion between them disrupted the interaction, the protein retained the closed conformation. Alternatively, in the four MD simulations with Tyr188 protonated (MD_Acid_^PrTr1^ and MD_Acid_^PrTr2^ systems in Fig. 7), after few nanoseconds of simulation a drastic rearrangement of the protein was observed. During these simulations (Fig. 8), the distance between the center of masses of NI and NII subdomains increased from a value close to the length measured at the X-ray crystal structure of the sTf holoform (25.9 Å) [61], to a value significantly larger (29-30 Å), although not as large as the value found at the X-ray crystal structure of the apoform (31.6 Å) [60]. In order to identify predominant global motion during the simulation, a Principle Component Analysis (PCA) was performed. The PCA revealed that the hinge-bending is the main global motion in those simulations with Tyr188 protonated and that this motion leads to a partial opening of the protein. On the other hand, there was not any predominant global motions in the simulations with Tyr188 unprotonated. Therefore, all these results indicated that the metal release (or binding) process is a stepwise mechanism. Starting from the closed conformation of the metal-loaded protein, the first step would be the hinge-bending motion. This motion enables an access of solvent to the metal binding site. Once the metal is in a solvent accessible area, the release of the metal would be facilitated by the hinge-twist motion of the protein. This two-step mechanism was previously described by Grossmann et al [62] for the release of iron. The simulations also highlight the importance of residues coordinated in the second coordination shell, for instance, the MD simulations remark the importance of the conformational changes of the Arg124 in the metal release mechanism of sTf.

In general, the MD simulations carried out with Fe(III) and Al(III) showed the same global motion of the protein, which may indicate that the molecular mechanism of the metal release from protein is analogous for both cations. They also demonstrated that Tyr188 must be protonated prior to the cation release. Nevertheless, a number of differences were identified at the atomic level between the simulations of the Fe(III)-sTf and Al(III)-sTf. Some of these differences are due to the fact that the simulations explored different subspaces, and that not all of them leaded to the same final conformations. However, it must be taken into account that the entire system, including the cation, was modeled with a non-polarizable force field. This treatment does not allow any charge transfer between the cation and its ligands, and therefore the coordination mode of the metal must be considered with caution.

#### 2. QM/MM calculations

For the study of the specific coordination mode of a metal in the binding site of a protein, it is convenient to allow charge transfer between the metal and its ligands. This charge transfer is not possible with a standard force field; instead, a quantum method is necessary. However, due to its size, only a region of the protein can be treated by quantum methods. Therefore, a hybrid Quantum Mechanics/Molecular Mechanics (QM/MM) scheme was employed [68] to investigate the coordination mode of Al(III) and Fe(III) at the sTf binding site. The entire chemical system was divided into a quantum mechanics (QM) region comprising the metal ion together with the side chains of all residues in the first coordination sphere, (carbonate ion, His249, Asp63, Tyr95 and Tyr188), and a molecular mechanics (MM) region which included the rest of the system (the rest of the protein and solvation water molecules). First, in order to take into account the dynamics of the system, two independent QM/MM molecular dynamics simulations were carried out, considering the different pH conditions in serum, where the metal binds to sTf, and in the endosome, where it is released. In the simulations, the QM part was treated with the AM1 semiempirical method and the CHARMM27 force field to treat the MM part. Secondly, in order to have a more accurate description of the metal loaded complex, several structures were picked up from these simulations and optimized with high level QM/MM methods, in which the quantum part was treated with density functional theory. These optimizations were carried out with Al(III) and Fe(III), what allowed to analyze the differences in their binding sites. Finally, the interaction of the sTf binding site with Al(III) and Fe(III) was further analyzed in small cluster models optimized in gas phase. In this model system only the metal and its ligands were included. All these results provided a detailed description of the metal loaded complex in diferent pH environments, highlighting the differences and similarities between them.

During the MD simulation at acidic conditions His249, who gained a proton with respect to the MD_Phys_ system, left the metal coordination shell to be accommodated in the second coordination sphere. The DFT/MM optimizations, which were confirmed by subsequent model cluster calculations, carried out for the Al(III)-sTf and Fe(III)-sTf complexes indicated that the coordination modes of these two cations can be different after the leaving of His249: Al(III) adopts a distorted tetrahedral conformation where Tyr95, Tyr188, Asp63 and the carbonate ion are placed in the four positions; on the other hand, Fe(III) maintains an octahedral arrangement where the carbonate ion is bidentated and the free position left by His249 is now occupied by the second O atom of Asp63. These differences in the binding mode of the two cations are of high relevance. At this point, it is tentative to relate these differences on the binding mode with the controversy about the interaction of the Al(III) load sTf with TFR. While the interaction between TFR and the Fe(III)-sTf complex is well documented [72], contradictory results are found in the literature regarding the interaction between TFR and the Al(III)-load sTf[44, 46]. In a recent study [73], Sakajiri et al concluded that the Al(III)-sTf structure is a trade-off between the open conformation presented by the apo-sTf and the closed conformation of Fe(III)-sTf. Therefore, one can hypothesize that the different interaction modes of the Al(III)- and Fe(III)-bound transferrin with TFR may come from the different binding modes at acidic conditions observed, although further investigations are still required to validate this hypothesis.

## V. ALUMINIUM AND OXIDATIVE STRESS

A particular area of recent interest is the capacity of aluminium to promote oxidative stress in biological systems. This is surprising since aluminium is in principle a non-redox metal. Nonetheless, since the seminal work of Fridovich et al [74], it is well known that Al(III) can exert a significant pro-oxidant activity. An early hypothesis by Exley [75] established that central to this ability was the possibility of stabilization by Al(III) of a superoxide radical anion O_2^·-^_. This could eventually lead to the formation of various reactive oxygen species either by a direct pathway with formation of the ·OOH radical, either indirectly by influencing the redox equilibrium in the Fenton reaction. In this section, we give computational examples of these two behaviors. In the first example we characterize how an Al(III) mononuclear complex is able to stabilize a superoxide radical departing from various Al(III)-hydrolytic species, and we determine the effect that Al(III)-superoxide complexes could have in the promotion of Fenton reaction by reduction of Fe(III) to Fe(II). On the second example, we analyze the capacity of Al(III)-boehmite to stabilize a superoxide and form an ·OOH radical. In both cases, computational methods suggest that the pro-oxidant activity of aluminium is high and, therefore, they support earlier hypothesis.

### A. Aluminium mononuclear complexes and superoxide stabilization

The existence of an Al(III)-superoxide (O_2^·-^_) complex has been hypothesized [75] as a key species in the pro-oxidant activity of Al(III)[74]. In fact, experimentally it is observed that the stronger the interaction between a metal and a superoxide, the larger oxidant capability of the metal [76–79]. One practical way to determine the interaction strength of a metal to a superoxide is through the evaluation of the ESR g-tensor values. Fukuzumi et al. established that the binding strength between a metal and a superoxide can be measured experimentally by the deviation of the EPR g-tensor value (in particular the g_zz_ value) from the spin-free value (g_e_=2.0023). The energy splitting (▵E) of the p_g_ levels of O_2^·-^_ due to the interaction with the metal can be estimated from the tensor values by a simple relation g_zz_=g_e_ + 2λ▵E, under condition that ▵E >> λ, where λ is the spin-orbit coupling constant of oxygen which is known as 0.014 eV. The larger the interaction of superoxide with the metal, the larger splitting caused in the π_g_ levels.

In Table I, we can find the calculated ▵E values for the M^n+^O_2^·-^_ complexes at the CASPT2 level of theory. For those metals for which there are experimental values, there is an outstanding agreement between theoretical and experimental data. We observe that the larger the positive charge and the smaller the size of the metal ion, the larger the splitting of π_g_ levels, specifically Al(III) provokes the largest splitting, 1.11 eV. These trends are coherent with the results of Fukuzumi et al [76–78] and more recent work of Kinraide et al [79]. Similar results were obtained for microsolvated structures [10].

**Table I:** Energy splitting of the π_g_ levels of the superoxide ▵E (estimated from g-tensor value^80^), ionization potential (IP) of M^n+^O_2^·-^_ and electron affinity (EA) of M^n+^O_2_ in eV, calculated at CASPT2 level of theory.

M^n+^	▵E (eV)		IP (eV)	EA (eV)
	**CASPT2**	**Exp.**		
Na^+^	0.35	0.34	7.3	4.9
K^+^	0.31	-	6.8	4.1
Mg^2+^	0.65	0.65	15.6	13.5
Ca^2+^	0.56	0.58	13.9	11
Al^3+^	1.11	-	25.5	25.1

However is this interaction strong enough as to displace water/hydroxide ligands from aluminium first solvation layer? In other words, could aluminium form these species in a biological environment? To answer this question, one can calculate the thermodynamics of the corresponding substitution reactions of a water/hydroxide bound to aluminium by a superoxide. To do it so, we have considered the effect of the pH, by analyzing the substitution reactions for a variety of hydrolytic species. Based on our pK_a_ calculations, we used a protocol based on a cluster-continuum approach, where we included two specific solvent layers and bulk solvent effects were treated with a dielectric continuum model. The protocol was also tested against pK_a_ values for HO_2_·.

The general trends are summarized in Table II. Irrespective of the hydrolytic species considered, the displacement of a water molecule from the first solvation layer around aluminium is always favorable, especially from Al(H_2_O)_6_^3+^ and Al(OH)(H_2_O)_5_^2+^ complexes. On the contrary, displacement of a hydroxide molecule is always endoergonic, and therefore will not take place. That is, the presence of Al(III) in an aqueous environment will lead to a significant stabilization of a superoxide through the formation of an Al(III)-superoxide complex. Once an aluminium-superoxide is formed this complex could influence the oxidative stress in biological systems in various ways. One of the possibilities is to increase indirectly the presence of reactive oxygen species, through the promotion of Fenton reaction, by enhancing the concentration of Fe(II), which in turn can reduce H_2_O_2_, provoking its breakdown and the formation of ·OH radicals. The question that arises is whether aluminium stabilization of O_2^·-^_ is so efficient that prevents from electron transfer to Fe(III). In this sense, we have evaluated the change in free energies for the redox reaction corresponding to an electron transfer from an aluminium-superoxide complex to Fe(III). Several theoretical approaches were used [11] in the context of cluster-continuum models, on the one hand, wave-function methods such as CASSCF and CASPT2, and on the other hand, several functionals within DFT level of theory, B3LYP, PBE, and M062X were used, all of them gave qualitatively similar results. For the sake of complementarity with other parts of this review, we only show the results obtained at B3LYP level of theory (Table II). A first result of our studies was the spontaneous release of the triplet molecular oxygen formed from the superoxide upon electron transfer.

**Table II:** B3LYP reaction free energies in kcal/mol, using two different continuum models, SMD and PCM. ΔG_g_ is obtained ΔG_aq_^X^ is calculated as ΔG_g_ + ΔΔG_solv_^X^ (X = PCM, SMD). The models included explicit first and second shell water molecules, except for the cases specified with a *, which contains only a first coordination sphere. For details see ref [10] and [11].

		SMD	PCM
**Al-superoxide Formation Reactions**			
Al(H_2_O)_6_^3+^+O·_2_^-^	Al(O·_2_)(H_2_O)_5_^2+^ + H_2_O	-8.3	-15.2
Al(OH)(H_2_O)_5_^2+^ + O·_2_^-^	Al(O·_2_)(OH)(H_2_O)_4_^+^ + H_2_O	-8.7	-13.5
	Al(O·_2_)(H_2_O)_5_^2+^ + OH^-^	13.5*	11.6*
Al(OH)_2_(H_2_O)_4_^+^ + O·_2_^-^	Al(O·_2_)(OH)_2_(H_2_O)_3_+H_2_O	-1.7	-2.8
	Al(·O_2_)(OH)(H_2_O)_4_^+^ + OH^-^	11.8*	7.8*
Al(OH)_3_(H_2_O)_2_+O·_2_^-^	Al(O·_2_)(OH)_3_(H_2_O)^-^ + H_2_O	-2	-1.8
	Al(O·_2_)(OH)_2_(H_2_O)_2_ + OH^-^	12.3*	6.8*
Al(OH)_4_^-^ + O·_2_^-^	Al(O·_2_)(OH)_3_^-^ + OH^-^	15.8	11.2

**Al-superoxide and Fe Redox Reactions**			
Fe^3+^ + AlO_2_·^2+^	Fe^2+^ + Al^3+^ + O_2_	-19.8	-7.4
Fe^3+^ + Al(OH)O_2_·^+^	Fe^2+^ + Al(OH)^2+^+O_2_	-19.2	-9.2
Fe^3+^ + Al(OH)_2_O_2_·	Fe^2+^ + Al(OH)_2_^+^ + O_2_	-18.9	-12.1

Therefore, the redox reaction should be written: Fe^3+^ + AlO_2_·^2+^ → Fe^2+^ + Al^3+^ + O_2_. The results, irrespective of the method to calculate the electronic energy, and the method to consider bulk solvent effects, was clearly exoergonic. Similar data was obtained for other Al(III)-superoxide complexes. In summary, aluminium-superoxide complexes are able to reduce Fe(III) to Fe(II),provoking the release of the oxygen molecule and recovering the initial aluminium hydrolytic species. As a result of all the process, there is the formation of a Fe(II) that is able to generate radicals, with the recoveryof an initial aluminium hydrolytic species, ready again for superoxide stabilization. Our results can be summarized in Fig. 11, in the so-called aluminium Fenton reaction promotion cycle.

### B. Aluminium boehmite and reactive oxygen species

Boehmite nanoparticles are used as adjuvant for vaccines [81, 82], because it induces an inflammatory response. Adsorption of tumor necrosis factor TNF-alpha was identified on boehmite [83]. Boehmite has shown to be toxic after inhalation by rats [84], and in the aggregate form, boehmite has an inflammatory and a cytotoxic activity [85]. Moreover macrophage myofasciitis have been reported in humans, which has been attributed to the long stay of boehmite particles in the muscle [86].

As a result, boehmite must be considered as exhibiting a potential risk factor for health. In fact, alumina nano-particles exhibit an oxidative stress activity (see ref [87] and references therein), however, to the best of our knowledge, no precise mechanisms have been proposed at the atomistic molecular scale for the action of boehmite particles in the body. One possibility is that boehmite acts as an Al(III) reservoir, even though the solubility of boehmite at neutral pH is low [85], and the oxidative activity is activated through the Fenton reaction promotion cycle proposed in the previous section. Another possible mechanism is due to the nanoparticles surface reactivity itself, an aspect that we have recently explored [88], and that we summarize in this subsection.

**Figure 9:**
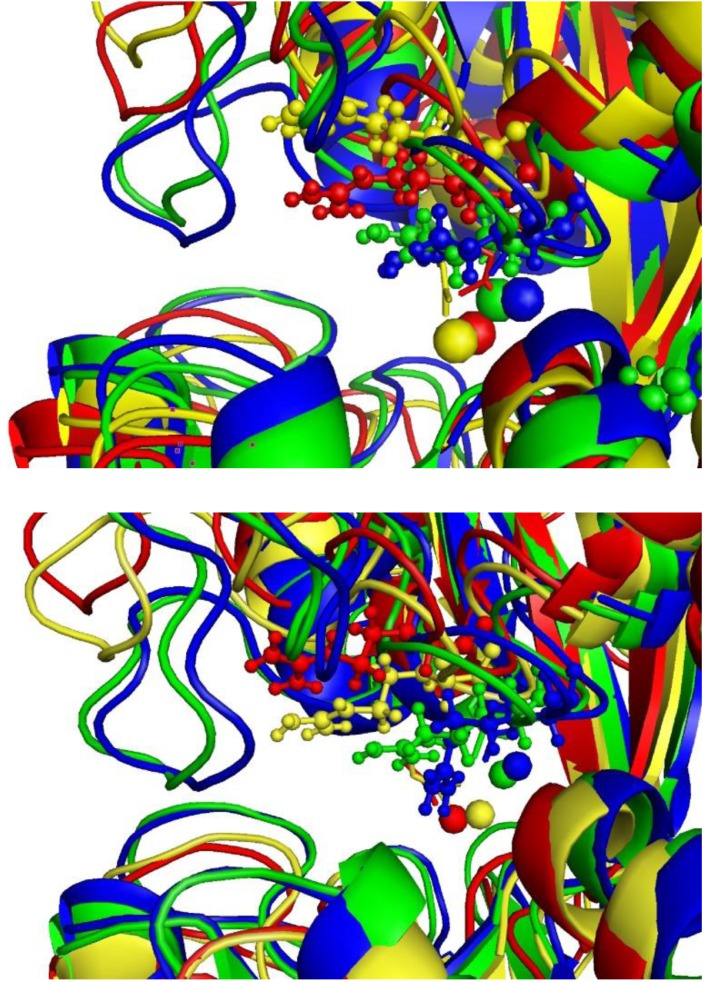
Superposition of representative snapshots of the molecular dynamics simulations with four protonation states considered for Fe(III)-sTf (on the top) and Al(III)-sTf (on the bottom): MD_Phys_ (in blue), MD_Acid_ (in green), MD_Acid_^PrTr1^ (in red) and MD_Acid_^PrTr2^ (in yellow). Arg124 is shown in ball and sticks and metal is in balls.

**Figure 10:**
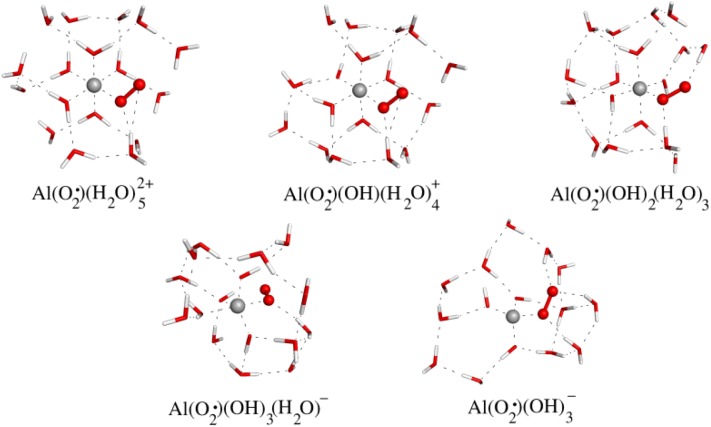
Al(III)-superoxide complexes of the type [Al(O_2_·)(H_2_O)_m_(OH)_n_]^(q-1)^, formed from [Al(H_2_O)_m_(OH)_n_]^q^ hydrolytic species, modeled using a cluster-continuum approach with two shells of explicit water molecules. Notice that thenumber of ligands in the first coordination shell changes as a function of the number of hydroxides in the first coordination sphere, i.e., from six in [Al(O_2_·)(H_2_O)_5_]^2+^ to four in [Al(O_2_·)(OH)_3_]^1-^.

**Figure 11:**
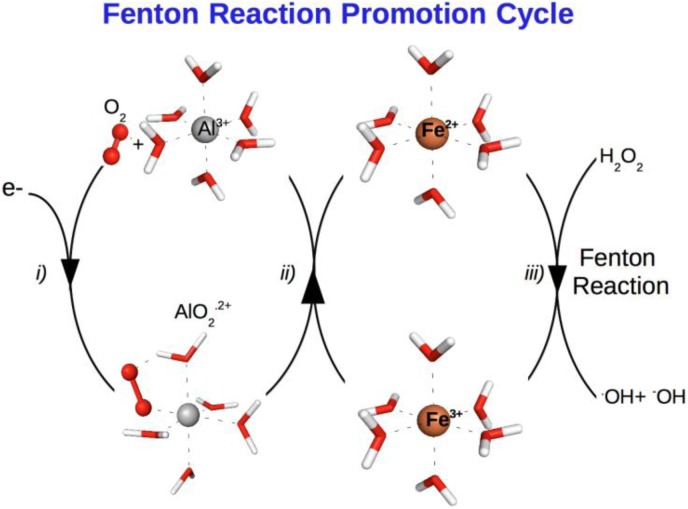
Aluminium can promote Fenton reaction through the following cycle: i) Aluminium is able to stabilize a superoxide radical anion O_2^·-^_, ii) The resultant Al(III)-superoxide complex is able to reduce Fe(III) to Fe(II), provoking the release of a neutral triplet O_2_ from the first solvation layer of aluminium, and thus recovering the initial aluminium hydrolytic species and iii) Fe(II) can induce the formation of ^·^OH radicals through the Fenton reaction. At the end of these steps we have generated reactive oxygen species that could trigger an important oxidative stress, recovering the initial aluminium hydrolytic species, which is ready to start again all the promotion cycle.

**Figure 12:**
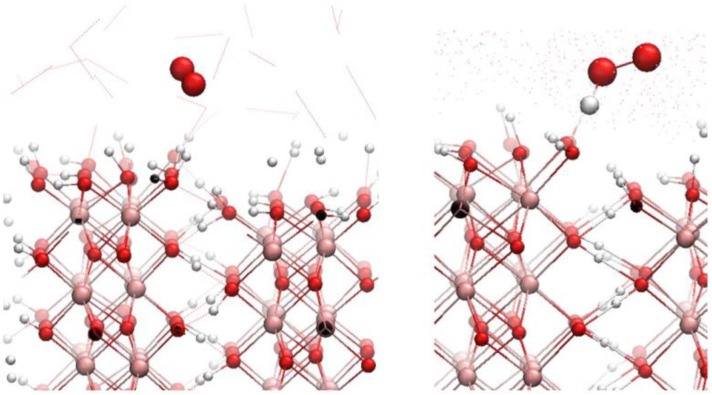
Systems envisaged for the study of the superoxide adsorption and reaction at the boehmite surface at the interface with water: on the left superoxide@surface, on the right OOH@surface.

We have performed DFT geometry optimizations and DFT-based molecular dynamics simulations related to the formation of the OOH· radical at the step of the boehmite surface (Fig. 12). We have considered the reactivity of a stepped boehmite surface towards the superoxide ion O_2^·-^_. Without the presence of surrounding water, we have shown that the superoxide anion adsorbs on the terrace, forming H bonds. On the step, O_2^·-^_ captures a proton, stabilizing an OOH· radical. The reaction is spontaneous without any activation energy. These tendencies were confirmed when considering the explicit presence of water solvent. We considered the superoxide anion at the boehmite step (Fig. 12 left) as well as the OOH· radical at the boehmite step (Fig. 12 right). The superoxide radical stabilized at 2.32 ± 0.02 Å from the surface, forming no H bond at the step. The configuration where one proton was abstracted from the surface and transferred to the superoxide radical, forming an OOH· radical is shown in Fig. 12. The OOH· radical forms a strong H-bond with the surface, where the surface is H-bond donor with O_OOH_ - H_surface_ = 1.50 ± 0.08 Å. The (OOH·@surface) configuration is more stable by -0.7 eV than the (superoxide@surface) configuration, suggesting a stabilization of the superoxide radical species in its protonated form at the surface. This result is explained by the acidic character of the µ2 - OH groups. A crude estimate of their acidic character of 3.7 can be made with the MUSIC model [89]. This pK_a_ is lower than the pK_a_ of superoxide (4.9) [10] and explains the proton transfer from the surface to the superoxide. This result is perfectly on line with previous result evidencing an increased proton conductivity at boehmite steps [90]. This protonation would enhance the oxidant ability of the resultant ROS, since OOH· radical is 104 times more oxidant than the superoxide anion. Stabilization of a very oxidative species might be of importance as this species might react with coadsorbed biomolecules, which are known to cover the inorganic surfaces once immersed in aqueous solution with biomolecules [91–93].

## VI. CONCLUSIONS

Biological systems have evolved in the absence of aluminium, the most abundant metal on the Earth crust. However, in the last century, several human actions have led to an increase in the bioavailability of aluminium. The presence of aluminium in our everyday life is so ubiquitous that the “aluminium age” expression has been coined by some authors to highlight the important exposition of our bodies to this metal. However, do we know the effects that such highly charged metal has in biological systems? In the last decades, consistent experimental evidences suggest that aluminium is not such as inert metal towards biosystems as it was thought. However, the understanding of aluminium speciation *in-vivo* and its effect in biological systems presents challenges from an experimental point of view. Theoretical methods have become in this sense a very important tool to shed light on aluminium chemistry, providing fundamental insight on its binding affinity, structures and potential toxic effects at the molecular level. In the present review, we have selected some examples of computational work done in this area, showing how different theoretical methods can be used to enhance our understanding of aluminium interaction with biomolecules.
